# Construction of a High Titer Infectious HIV-1 Subtype C Proviral Clone from South Africa

**DOI:** 10.3390/v4091830

**Published:** 2012-09-24

**Authors:** Graeme B. Jacobs, Stefanie Bock, Anita Schuch, Rebecca Moschall, Eva-Maria Schrom, Juliane Zahn, Christian Reuter, Wolfgang Preiser, Axel Rethwilm, Susan Engelbrecht, Thomas Kerkau, Jochen Bodem

**Affiliations:** 1 Institut für Virologie und Immunbiologie, Universität Würzburg, Versbacher Straße 7, 97078 Würzburg, Germany; Email: graeme@sun.ac.za (G.B.J.); bock-steffi@web.de (S.B.); anita.schuch@stud-mail.uni-wuerzburg.de (A.S.); rebecca.moschall@stud-mail.uni-wuerzburg.de (R.M.); eva-maria.schrom@stud-mail.uni-wuerzburg.de (E.-M.S.); juliane_zahn@gmx.de (J.Z.); christian.reu@web.de (C.R.); virologie@vim.uni-wuerzburg.de (A.R.); kerkau@mail.uni-wuerzburg.de (T.K.); 2 Division of Medical Virology, Faculty of Medicine & Health Sciences, Stellenbosch University/National Health Laboratory Service, Tygerberg, 7505, Cape Town, South Africa; Email: preiser@sun.ac.za (W.P.); susanen@sun.ac.za (S.E.)

**Keywords:** HIV-1, subtype C, proviral plasmid, viral replication, resistance assays, Vpu, CD317, CD4

## Abstract

The Human Immunodeficiency Virus type 1 (HIV-1) subtype C is currently the predominant subtype worldwide. Cell culture studies of Sub-Saharan African subtype C proviral plasmids are hampered by the low replication capacity of the resulting viruses, although viral loads in subtype C infected patients are as high as those from patients with subtype B. Here, we describe the sequencing and construction of a new HIV-1 subtype C proviral clone (pZAC), replicating more than one order of magnitude better than the previous subtype C plasmids. We identify the *env*-region for being the determinant for the higher viral titers and the pZAC Env to be M-tropic. This higher replication capacity does not lead to a higher cytotoxicity compared to previously described subtype C viruses. In addition, the pZAC Vpu is also shown to be able to down-regulate CD4, but fails to fully counteract CD317.

## 1. Introduction

The genetic subtype distribution of HIV-1 group M, currently responsible for the majority of the AIDS pandemic, has become dynamic. The HIV-1 group M has been divided into nine subtypes, 51 circulating recombinant forms and numerous unique recombinant forms. From 2004–2007, subtype C accounted for nearly half (48%) of all global infections [1]. HIV-1 subtype C is predominant in Eastern and Southern Africa as well as in India. It is responsible for approximately 95% of all HIV-1 infections in Southern Africa [2] and is increasing in frequency in countries such as China and Brazil [1] showing that subtype C viruses have high transmission efficiency *in vivo*. HIV-1 subtype C has unique characteristics: These include the presence of an additional NF-κB enhancer binding site in the long terminal repeat (LTR), a prematurely truncated Rev, and an insertion of five amino acids at the amino terminus of Vpu [3,4]. 

There are currently five HIV-1 subtype C proviral clones described, two from Sub-Saharan Africa [5,6], two from India [7,8] and one chemically synthesized proviral plasmid of Indian sequence origin [9]. Viral loads observed in ARV naïve patients infected with HIV-1 subtype C are in the same range of subtype B isolates [10,11]. Curiously, subtype C molecular clones such as pMJ4 show significant lower replication-fitness in primary CD4^+^ T cells and peripheral blood mononuclear cells (PBMCs) compared to subtype B clones, such as pNL4-3 [6,12,13]. 

## 2. Results and Discussion

**Figure 1:**
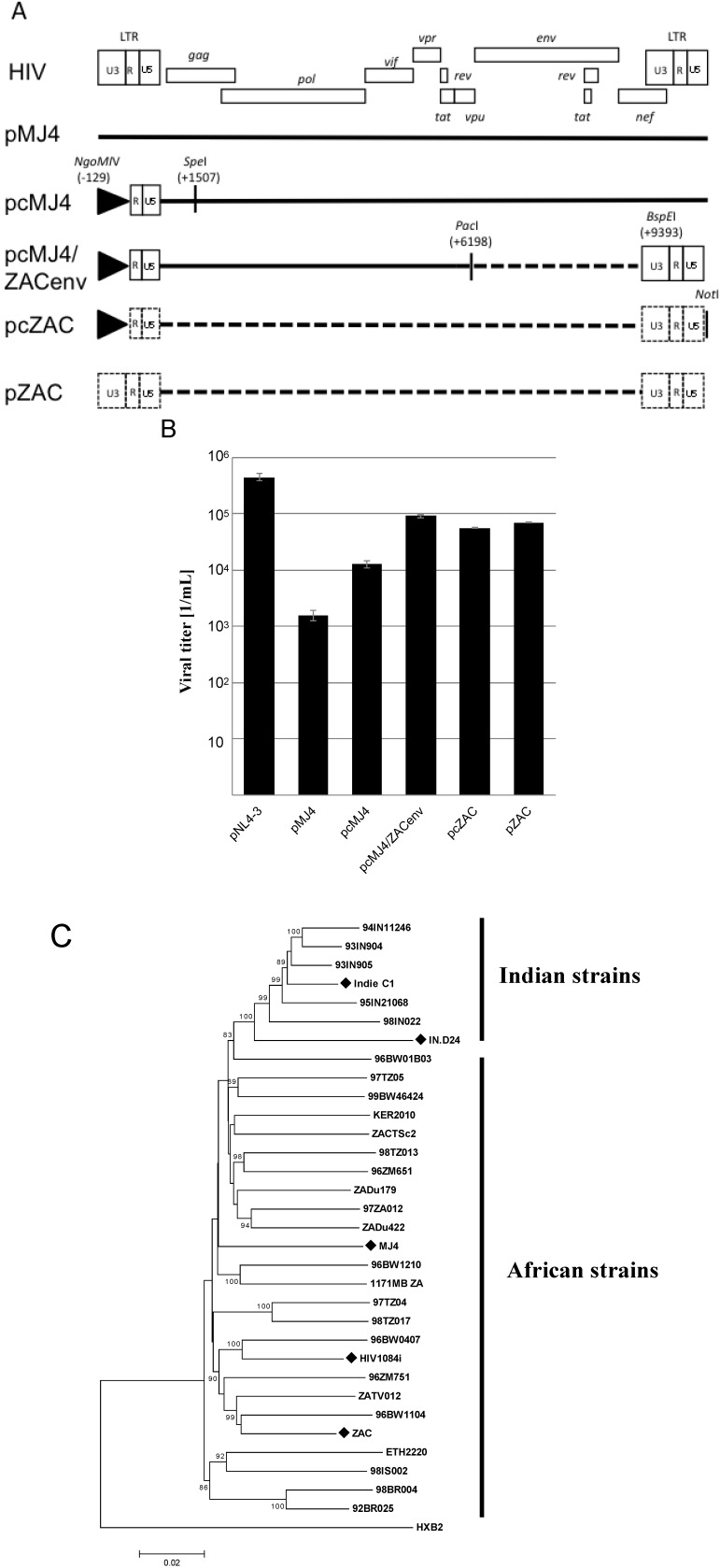
Molecular characterization of pZAC, an infectious proviral clone from Cape Town, South Africa. (**A**) Cloning strategy used during the study. The U3 promoter of pMJ4 was replaced with a CMV-IE promoter, as indicated. The CMV-promoter is represented by a triangle. The proviral clone pcMJ4 was used as a backbone for further characterization of pZAC. Dotted lines indicate patient ZAC derived sequences. The enzyme restriction sites used for cloning are indicated.(**B**) Transient virus titer on TZM-bl of infectious proviral clones. After transfection of HEK 293T cells, cultured supernatants were titrated on HeLa TZM-bl indicator cells to determine the transient viral titer of HIV proviral clones [16]. Titers and standard deviation were derived from three independent experiments. (**C**) Phylogenetic analysis of HIV-1 subtype C infectious clones. A Neighbour-Joining tree was drawn from the infectious HIV-1 subtype C clones, compared to a HIV-1 subtype C reference set (dataset obtained from [17]). Evolutionary distances were calculated using the Maximum Composite Likelihood method, with a bootstrap test of 10,000 replicates. The branch scale, indicating the evolutionary distance, is indicated. The Indian and African strains form two unique phylogenetic clusters, with the newly described pZAC sequence showing similarity to the Botswana HIV-1 subtype C sequences, 96BW1104. The HIV-1 subtype B reference sequence, HXB2 was used as an outlier to root the phylogenetic tree.

We sought to establish a subtype C proviral plasmid, which gives rise to viral loads comparable to those seen with subtype B proviral plasmids. The cloning strategy was based on the existing pMJ4 plasmid, where fragments were replaced with patient derived HIV-1 subtype C sequences. For this DNA isolated from the HIV-1 positive patient R3714/ZAC was used [14]. Patient ZAC is one of the earliest (1989) documented cases of heterosexual transmission of HIV-1 subtype C in South Africa [14]. We first replaced the *env* region of pMJ4 with that of our primary isolate (Figure 1A). The pMJ4 proviral plasmid was chosen, since the partial ZAC sequences indicated a higher homology to pMJ4 compared to the other proviral clones. Transfection of HEK 293T cells with pcMJ4/ZAC env showed an approximately 10fold increase in viral titer compared to pMJ4 (Figure 1B).

Both the *gagpol* and LTR-*gag* regions were amplified and used to replace the corresponding pMJ4 sequences (Figure 1C, supplementary materials). The new proviral plasmid was designated pZAC. This plasmid is free of any pMJ4-derived sequences. The pZAC sequence shows the typical African HIV-1 subtype C characteristics mentioned above (3 Nf-κB sites, premature *rev* stop codon, 5 amino acid insertion in Vpu). The phylogenetic relationships of the full-length infectious HIV-1 subtype C clones were analysed by constructing a neighbour-joining tree (Figure 1C) [15]. The Indian and African HIV-1 subtype C strains exhibited two unique phylogenetic clusters (Figure 1C). 

**Figure 2:**
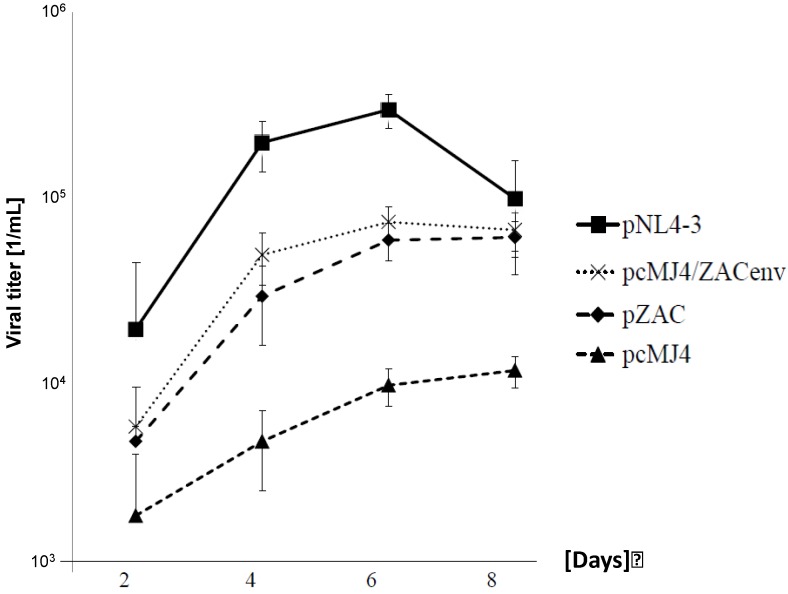
pZAC replicates faster in PBMC than pcMJ4. Growth kinetics of subtype B and C viruses on PBMCs. Infectious viruses (MOI: 0.05) were cultured for 8 days on PBMCs. The supernatants were titrated on TZM-bl cells to determine the viral titer as indicated. Experiments were done in triplicate. NL4-3 had the highest replication capacity, whereas pMJ4/ZACenv and pZAC replicated better than pcMJ4. pZAC and pcZAC has similar growth kinetics, whereas pMJ4 failed to show significant viral titers after 8 days of culture.

To analyse replication kinetics, the infectious viruses (multiplicity of infection (MOI) of 0.05) were cultivated on PBMCs for up to 8 days (Figure 2). Viral titers were determined on TZM-bl cells. Although pcMJ4/ZACenv and pZAC replicated significantly better in PBMCs than the pMJ4 virus, NL4-3 titer levels were never reached. The latter is in-line with the results obtained with primary isolates [18]. pZAC and pcMJ4/ZACenv infectivity peaked at days 4–6, similarly as described for the Indian HIV-1 subtype C [7,8]. 

It has been reported that the HIV subtype C displays a lower cytopathic effect than other subtypes. Since our cloned virus replicates at a higher titer than the previously characterised pMJ4, we analysed whether the low cytopathic effect was conserved with pZAC. To determine cell damage and elimination cause by pMJ4 and pZAC viruses, TZM-bl cells were infected with a MOI of 0.25 in triplicate assays. The cell survival and cellular metabolism was measured after two days by a cell proliferation assay according to the manufacturers instructions. The MTT values of both viruses were found to be in a similar range (ZAC, OD_490_ = 1.48 ± 0.049 pMJ4, OD_490_ = 1.32 ± 0.05; uninfected cells, OD_490_ = 1.36 ± 0.1). This experiment demonstrated that the infectious subtype C viruses did not affect the cellular metabolism in a significant way.

**Figure 3:**
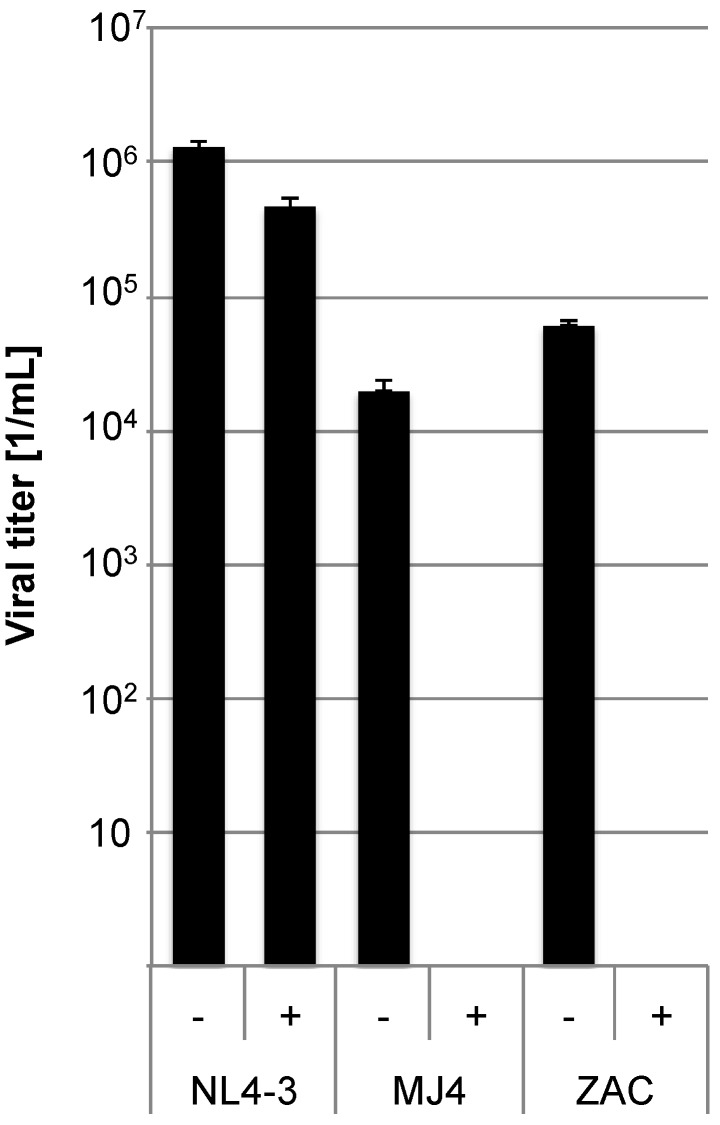
CCR5 is the coreceptor used by both pMJ4 and pZAC. In order to determine the co-receptor usage TZM-bl cells were pre-incubated with 10 ng/mL of the CCR5 inhibitor Maraviroc (indicated by (+)) for 1 h and subsequently infected with NL4-3, pMJ4 and pZAC viruses (NL4-3 MOI: 2; MJ4 and ZAC MOI: 0.2). Untreated control cultures are indicated by (−). Infected cells were visualized by X-Gal stain and counted. Both pMJ4 and pZAC use CCR5 as coreceptor. Experiments were performed in independent triplicates and the experiment was repeated twice. Error bars indicate the standard deviation within one experiment.

Since a determinant for the higher pZAC titers is located in the *env* region, we sought to analyse this in detail (supplementary materials). We determined the co-receptor usage to exclude that it accounts for the differences in replication capacity between pMJ4 and pZAC. Viruses were produced by transfecting HEK 293T cells with the pZAC, pMJ4, or pNL4-3 plasmids and the titers were subsequently determined. TZM-bl target cells (2 × 10^4^) were pre-incubated with 10 ng/mL of the CCR5 inhibitor Maraviroc for one hour. Cell culture supernatants were titrated on these target cells (MOI: 2) (Figure 3). The T-tropic NL4-3 was used as control. Both pMJ4 and pZAC were completely inhibited by Maraviroc, whereas the NL4-3 virus was unaffected, showing that pMJ4 and pZAC only use CCR5.

**Figure 4:**
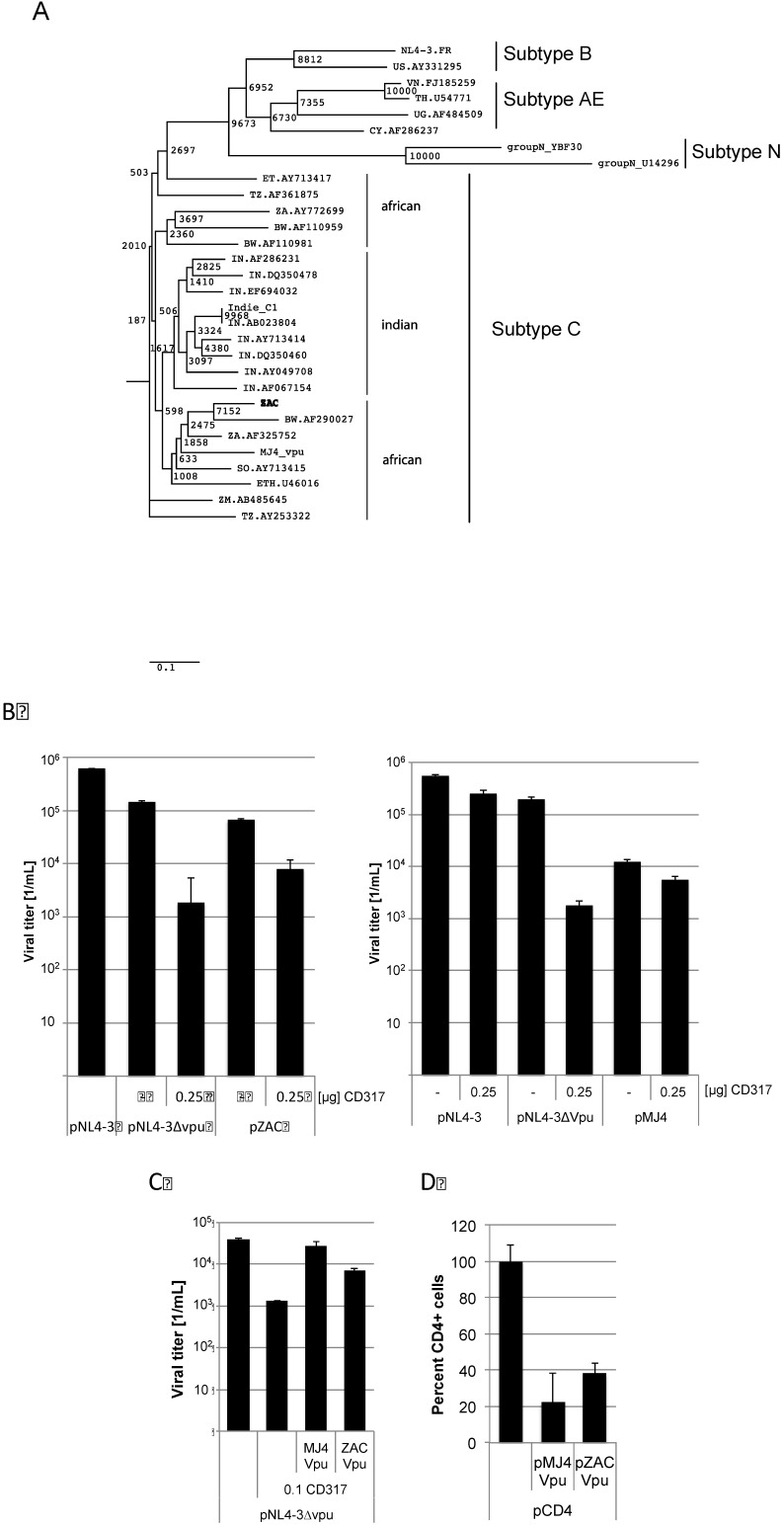
pZAC Vpu is unable to counteract CD317 but down-regulates CD4 expression at the cytoplasmic membrane. (**A**) Phylogenetic tree of the Vpu amino acid sequences. Evolutionary distances were calculated using the Maximum Composite Likelihood method, with a bootstrap test of 10,000 replicates. The branch scale, indicating the evolutionary distance, is indicated. The African subtypes do not form a unique cluster with the newly described pZAC sequence. Subtypes N, B, and AE were used as out-group. (**B**) pZAC is not completely able to counteract CD317. HEK 293T cells were co-transfected with pNL4‑3∆vpu, pZAC, or pMJ4 and 0.25 µg of pCD317. Cellular supernatants were titrated on TZM-bl cells and viral titers were determined. Experiments were performed in triplicate. Error bars indicate the standard deviation. (**C**) The pZAC Vpu is unable to rescue CD317 sensitivity of pNL4-3∆vpu to wild type levels. MJ4 Vpu is significantly better at antagonising CD317 than that of ZAC Vpu. HEK 293T cells were transfected with pNL4-3∆vpu and pZAC *vpu* or pMJ4 *vpu*. Viral titers were determined on TZM-bl cells. Bars represent the mean of 3 independent experiments. (**D**) pZAC and pMJ4 are able to down-regulate CD4 expression in a similar way. HEK 293T cells were cotransfected with the CD4 expression construct pMSCCD4 and pZAC *vpu* or pMJ4 *vpu*. CD4 expression was monitored by FACS. CD4 expression of the control without *vpu* was set to 100%. Bars represent the mean of 3 independent experiments. Error bars indicate the standard deviation.

By analysing the pZAC sequence we discovered a divergent evolution of the Vpu amino acid sequences compared to the complete viruses (Figure 4A). The African Vpu sequences form two distinct clusters. Both pZAC and pMJ4 encode the 5 amino acid insertion in Vpu. Since Vpu is encoded in the *env* fragment exchanged in the pMJ4/ZAC clone, we analysed Vpu function to antagonize CD317. CD317 was shown to inhibit virus release [19]. The proviral plasmid of pZAC and pMJ4 were co-transfected with 0.25 µg of pCD317. A *vpu*-ATG deficient pNL4-3∆vpu served as control. Viral titers were determined by titration on TZM-bl cells 2 days post transfection. pZAC derived titers were significantly reduced by CD317 indicating that pZAC Vpu is unable to significantly counteract CD317 (significance of the titer reduction as calculated by the paired two-sample t-test *p* = 0.009), while pMJ4 titers remained unaffected (significance of the titer reduction as calculated by the paired two-sample t-test *p* = 0.07) (Figure 4B). In order to exclude indirect factors, experiments using pZAC or pMJ4 Vpu to rescue NL4-3∆vpu release were conducted. The *vpu* sequences of pMJ4 and pZAC were cloned in pcDNA3.1. HEK 293T cells were cotransfected with 2.4 µg of the proviral HIV plasmids pNL4-3∆vpu [20] and pMJ4 *vpu* (3 µg) or pZAC *vpu* (3 µg) (Figure 4C). Viral titers were measured on TZM-bl cells as described above. CD317 reduced the viral titer about 1.5 orders of magnitude. pMJ4 Vpu rescued this titer reduction (significance of the titer reduction as calculated by the paired two-sample t-test *p* = 0.09), whereas pZAC Vpu failed to counteract CD317 completely (significance of the titer reduction as calculated by the paired two-sample t-test *p* = 0.006). These results show that the ability of pZAC Vpu to counteract CD317 is impaired compared to pMJ4 Vpu.

Vpu was reported to down-regulate CD4 as well as counteract the effects of CD317 [21,22,23]. It has been shown previously that HIV-1 subtype B Vpu has different biological properties than subtype C Vpu. Although subtype C Vpu proteins are more efficiently transported to the cell surface, it is less efficient at down-regulating CD4 than the subtype B Vpu [24]. We sought to analyse whether CD317 counteracting activity was conserved in the pZAC Vpu or was decreased in the same way as previously described for subtype B Vpu. HEK 293T cells were cotransfected with pMSCVCD4, peGFP and pZAC Vpu or pMJ4 Vpu. Cells were harvested 4 days after transfection and stained for CD4 expression at the cytoplasmic membrane with a PerCP-coupled anti-CD4 antibody (Figure 4D). Surface CD4 expression was determined by FACS. pMJ4 Vpu and pZAC Vpu down-regulated CD4 expression significantly (Figure 4D). This indicates that CD4 down-regulation was still required in the patient ZAC for replication, while efficient CD317 counteraction seemed to be dispensable.

The surprising result of this study was that the pZAC Vpu fails to fully counteract CD317, which might be an immanent problem of other subtype C viruses as well. By analysing patient derived sequences from South Africa, we have observed that about 1% of these viruses showed a deletion in the *vpu* start codon. Functional analysis of one of these patient derived sequences showed that no functional Vpu was expressed (data not shown). So far only HIV-1 subtype N and P have been shown not to counteract CD317 by Vpu [25,26]. Unfortunately it is unknown whether these viruses are M- or T-tropic, since only macrophages were shown to express CD317 at high levels [27]. The virus infections of PBMCs showed that viruses encoding the pZAC *env* region were able to grow. The same holds true for the pcMJ4/ZAC*env*, which expresses only the *env*-region of pZAC in a pMJ4 background. These data raise the question, whether pZAC titers would further increase if the vpu sequence were replaced by sequences from ZAC.

## 3. Experimental Section

**Patient data:** The retrovirus cohort represents patient samples diagnosed with either HIV or HTLV infection of which samples (plasma and serum) were stored from 1984 to 1995 at the Tygerberg hospital in Cape Town, South Africa. The patient, a South African coloured (mixed race) male born on 22 August 1931 was diagnosed with lymphocyte depleted Hodgkin’s lymphoma on 02 March 1989 and diagnosed as HIV-1 positive on 09 March 1989. He travelled frequently to Lusaka, Zambia, where he possibly became infected with the virus. 

Subsequently, serum and peripheral blood mononuclear cells (PBMCs) were obtained during November 1989 (harvested on 20 and 21 November 1989) and the virus was co-cultured with PBMCs and isolated. High molecular weight DNA was extracted from the HIV positive cultures through phenol-chloroform extraction and stored. HIV-1 positive cultures were confirmed by reverse transcriptase (RT) assay that ranged from 12,495 to 35,073 counts per minute per milliliter (cpm/mL). The *env* gene was amplified by PCR, sequenced and identified as subtype C [14].

**Exchange of the 5'-U3 promoter with CMV-IE-promoter:** All PCR amplifications during this study were done with the stable proofreading Herculase II polymerase (Stratagene). Briefly, the HIV-1 subtype C promoter was replaced and cloned into the pMJ4 vector by overlapping PCR, using restriction sites (Figure 1). The 600 bp CMV promoter region was amplified from pEGFP-C1 with primers CMVstart_NgoMIV (5'-GAATGCCGGCTAGTTATTAATAGTAATCAATTACGGGTC-3'), containing a *NgoM*IV restriction site, underlined in the sequence and CMV_overlap_R (5'-GTGCTCCCGGGCTCAGATCTGGTCTACCTAGAGAGACCCGGTTACTAAACCAGCTCTG-3'), containing the last 30 bp of the CMV promoter and the first 30 bp from HIV-1 subtype C transcription start (R-region). The 1.0 kb HIV-1 subtype C region from R-start to the *Spe*I site in *gag* was amplified from pMJ4 with primers CMF_overlap_F (5'-CAGAGCTGGTTTAGTAACCGGGTCTCTCTAGGTAGACCAGATCTGAGCCCGGGAGCTC-3') and HIVC_SpeI_R (5'-CTATTTGTTCCTGAAGGGTACTAGTGTTCCTGCTATG-3'), the *Spe*I site is underlined. Primers CMVstart_NgoMIV and HIVC_SpeI_R were then used to PCR amplify the 1.6 kb fragment and cloned directly into pMJ4. The presence of the CMV promoter was confirmed by DNA sequencing. The resulting plasmid was abbreviated as pcMJ4.

**Cloning of pZAC:** The cloning strategy used during this study is described in Figure 1A. We first replaced the *env* of pMJ4 with that of our primary isolate, ZAC using standard cloning techniques. The 3.2 kb PCR product was amplified from the HMW DNA of the patient with primers containing the restriction enzyme recognition sites for *Pac*I and *BspE*I (for primer sequences see supplementary materials). This corresponds to position 6198 and 9393 relative to the reference HXB2 genome. Clones were screened by restriction enzyme digestion and sequenced to confirm the presence of the correct insert. The 5’ fragment of pZAC was amplified in two further parts encompassing the *gagpol* and LTR-*gag* region. The *gagpol* region was replaced using restriction sites *Spe*I (corresponding to position 1507 of HXB2) and *Pac*I (corresponding to position 6198 of HXB2), while the CMV-IE LTR-*gag* sequence was added as for pcMJ4. The new proviral clone was designated pcZAC. The 3'‑U5 was replaced using *BspE*I and the vector located *Not*I restriction site and the 5'-U3 CMV-IE was replaced with the pZAC derived 5'-U3 sequence. The final clone (without the CMV-IE promoter) was named pZAC. The pZAC sequence was submitted to GenBank: Acc. No. JN188292.

**Sequence and phylogenetic analyses:** Full-length HIV-1 genome sequencing was done with a set of primers previously described [28]. Sequence and phylogenetic analyses were done with previously described methods as well [29]. Phylogenetic trees were generated with the Mega version 5 software package [15].

**Isolation of PBMCs and maintenance for cell cultures:** PBMCs were extracted from donor EDTA blood by density gradient centrifugation with Histopaque^®^-1077 (Sigma-Aldrich). PBMCs were cultivated overnight at 37 °C at 5% CO_2_ with RPMI-1640 media stimulated with phytohemagglutinin-P, (PHA-P) (Sigma-Aldrich) (0.5 mg/mL) and human Interleukin-2 (IL-2) (Sigma‑Aldrich) (0.1 mg/mL) before being used for viral kinetics assays. HEK 293T and TZM-bl cells were maintained in minimal essential media (MEM) (Invitrogen) at 37 °C at 5% CO_2_. Cellular metabolism and cell viabilty was detemined with the cell proliferation assay (Promega) 2 d post infection. In brief: 20 µL of the indicator solution was added to 100 µL cultures. The cells were further incubated in the cell culture incubator for 90 min. The reaction was stopped by the addition of 25 µL 10% SDS and the absorbance was measured at 490nm. Uninfected cells served as control. 

**Transfection of cell lines:** For the infectious clones approximately 4 μg of plasmid DNA was transfected with the Fugene^®^6 transfection reagent (Roche Diagnostics) and the culture incubated for 2 to 3 days to allow for the expression of proteins.

**Infectivity assays:** To analyse the infectivity cell culture supernatants were titrated on TZM-bl cells in triplicate assays as described before [16]. In brief: cell culture supernatants were collected, centrifuged (1,500 rpm, 5 min) to remove infected cells and titrated in serial dilutions on TZM-bl cell (96-well plate, 104 cells per well). TZM-bl cells carry a stably integrated copy of a HIV LTR promoter driving a *lacZ* gene. Two days post infection TZM-bl cells were fixed with ice-cold methanol/acetone for 5 min followed by a β-galactosidase stain, using X-gal as substrate. Blue cells were counted and the viral titers were calculated. The number of GFP expressing cells was determined after two days using a fluorescence microscope. Viral titers were subsequently calculated. A paired two-sample t-test was used to obtain the p-values given in the manuscript.

**CD4 determination: **HEK 293T cells were cotransfected with the 2 µg of pMSCCD4 expression, 0.5 µg peGFP and 2 µg of the pZACvpu or pMJ4vpu expression plasmids. Cells were harvested and washed with FACS buffer. The monoclonal anti-human CD4 antibody (BioLegend) was diluted 1:25 and cells were stained for 15 min at 4 °C. The cells were subsequently fixed with Fix & Perm reagent (Invitrogen). CD4 expression was measured by FACS. CD4 expression of the transfected GFP expressing cells was analysed using the FlowJo software [30].

## 4. Conclusions

HIV-1 subtype C remains the predominate subtype worldwide and is especially prevalent in Sub‑Saharan Africa, where the HIV/AIDS epidemic is highest. However, comparative *in vitro* studies with proviral HIV subtype B strains have shown that HIV-1 subtype C generally has a lower replication rate [5,6]. This is not correlated with *in vivo* titers of infected patients, where similar viral loads are observed. We show here that the exchange of the *env* region of the proviral pMJ4 plasmid enhances infectivity. We have shown recently that the usage of the authentic subtype is required especially for the analysis of resistance mutations, such as a highly mutated proteases, since the viral background is necessary for fitness compensation [31]. The pZAC plasmid will help to perform phenotypic resistance assays with subtype C, due to its high replication capacity. Analysis of the ZAC Vpu revealed that it supported CD4 down-regulation, whereas it failed to antagonize CD317. This indicates that CD4 down-regulation was still required in the patient ZAC for replication, while CD317 counteraction seemed to be dispensable. 

## Supplementary Files

Supplementary File 1: PDF-Document (PDF, 567 KB)
